# Expectant parents´ perceptions about the grandparents’ role on their transition to parenthood: an exploratory study

**DOI:** 10.1192/j.eurpsy.2026.11942

**Published:** 2026-07-31

**Authors:** I. M. M. M. D. Mendes, A. F. F. Silveiro, S. M. Coelho, R. M. C. Rodrigues

**Affiliations:** Nursing School of Coimbra, Coimbra, Portugal

## Abstract

**Introduction:**

The transition to parenthood is a process that involves a series of changes affecting not only the infant’s parents but also the entire family. Scientific evidence highlights grandparents as the primary support and assistance network for the family during this process.

**Objectives:**

To identify the perceptions of expectant parents regarding expectations, concerns and involvement of grandparents in their transition to parenthood.

**Methods:**

Qualitative exploratory-descriptive study, using a non-probability snowball sample of 31 expectant parents, after obtaining informed consent. Data collection conducted through an online questionnaire after the study received ethical approval from the Ethics Committee of the Research Unit. Data analyzed using content analysis according to Bardin (2016).

**Results:**

The analysis of data revealed three key dimensions: *expectations regarding grandparents’ involvement*; *tensions and differences in the relationship with maternal and paternal grandparents*; and *relationship and influence of grandparents on parenting* (encompassing the quality of the relationship, the influence perceived by the couple, and the fundamental elements of a healthy intergenerational relationship).

**Conclusions:**

Based on the perceptions of expecting parents, the role of grandparents is not limited to practical support. It encompasses the establishment of a balanced emotional relationship, in which mutual respect and collaboration facilitate the transition to parenthood. The relationship between parents and grandparents it´s strongly influenced by the nature of the pre-existing bond and, by how this relationship it´s managed during the transition to the new parental role in respect to intergenerational relationship.

**Disclosure of Interest:**

None Declared

